# A proposed approach in defining population-based rates of major injury from a trauma registry dataset: Delineation of hospital catchment areas (I)

**DOI:** 10.1186/1472-6963-8-80

**Published:** 2008-04-10

**Authors:** Roxana Alexandrescu, Sarah J O'Brien, Ronan A Lyons, Fiona E Lecky, the Trauma Audit and Research Network

**Affiliations:** 1 Trauma Audit and Research Network, Clinical Science Building, Hope Hospital, University of Manchester, Eccles Old Road, Salford, M6 8HD, UK; 2 School of Translational Medicine, Clinical Science Building, Hope Hospital, University of Manchester, Eccles Old Road, Salford, M6 8HD, UK; 3 Centre for Health Information, Research and Evaluation (CHIRAL), School of Medicine, Swansea University, Singleton Park, Swansea, SA2 8PP, Wales; 4 Trauma Audit and Research Network, Clinical Science Building, Hope Hospital, University of Manchester, Eccles Old Road, Salford, M6 8HD, UK

## Abstract

**Background:**

Determining population-based rates for major injury poses methodological challenges. We used hospital discharge data over a 10-year period (1996–2005) from a national trauma registry, the Trauma Audit and Research Network (TARN) Manchester, to construct valid numerators and denominators so that we can calculate population-based rates of major injury in the future.

**Methods:**

We examined data from all hospitals reporting to TARN for continuity of numerator reporting; rates of completeness for patient postcodes, and clear denominator populations. We defined local market areas (>70% of patients originating from the same postcode district as the hospital). For relevant hospitals we assessed data quality: consistency of reporting, completeness of patient postcodes and for one selected hospital, North Staffordshire Royal Infirmary (NSRI), the capture rate of numerator data reporting. We used an established method based on patient flow to delineate market areas from hospitals discharges. We then assessed the potential competitors, and characterized these denominator areas. Finally we performed a denominator sensitivity analysis using a patient origin matrix based on Hospital Episodes Statistics (HES) to validate our approach.

**Results:**

Sixteen hospitals met the data quality and patient flow criteria for numerator and denominator data, representing 12 hospital catchment areas across England. Data quality issues included fluctuations numbers of reported cases and poor completion of postcodes for some years. We found an overall numerator capture rate of 83.5% for the NSRI. In total we used 40,543 admissions to delineate hospital catchment areas. An average of 3.5 potential hospital competitors and 15.2 postcode districts per area were obtained. The patient origin matrix for NSRI confirmed the accuracy of the denominator/hospital catchment area from the patient flow analysis.

**Conclusion:**

Large national trauma registries, including TARN, hold suitable data for determining population-based injury rates. Patient postcodes from hospital discharge allow identification of denominator populations using a market area approach.

## Background

In the United Kingdom, injury is the commonest cause of death in the first four decades of life and amongst the leading causes for ill-health. In 2004 in England and Wales 17,000 deaths due to injury (all causes) were registered. These represent only the tip of the 'injury pyramid', best described as: 'for every injury death there are 45 hospitals episodes, 630 doctor consultations and 5000–6000 minor injuries' [1]. Although the Government has a target to reduce deaths from accidents by at least a fifth and to reduce serious injury rates from accidents by at least a tenth by 2010 [2], injury remains a 'neglected epidemic' especially in relation to research and prevention.

Most official injury statistics and research studies in UK focus on mortality data [3,4], often used as a substitute for injury rates. Morbidity information at regional or national level comes from health and general household surveys, morbidity surveys in general practice [5-8] and from research papers. Population-based injury rates are mainly from research articles covering large, well-defined geographical populations in the Midlands and Wales [9,10]. A major drawback of these studies is that they cover limited periods of time and fail to account for injury severity in a standardised way. Consequently UK population-based incidence rates of serious injury are currently unknown. Trauma registries, and in particular the Trauma Audit and Research Network (TARN), have the potential to provide timely information on injury occurrence rates categorised by an internationally recognised specific injury scoring method, namely Injury Severity Score (ISS), that accounts for major injury (i.e. injury defined by ISS>15) [11]. These data could, therefore, be used to guide priorities for injury control and allow international comparisons. However membership of TARN is voluntary within England and Wales and currently limited to a 50% sample of hospitals receiving trauma cases.

This is the first in a series of articles. In this article we propose to determine whether hospital based injury data from a large (but not comprehensive) trauma registry can be used to derive population based rates of major injury. To do this we examine the potential of a patient flow-based method for defining hospitals' catchment areas, which is practical for calculating denominator populations. We select a number of hospitals from the TARN database taking into account data quality and pattern of admissions, delineate hospitals' catchment areas for these hospitals and characterized these areas. Finally we examine the sensitivity of these numerator and denominator populations using Hospital Episodes Statistics (HES) data. Later articles will look at the computation of injury rates by patients' demographic characteristics and injury details and at the extrapolation of rates to the national population.

## Methods

### Data Source

#### (1) Patient data

The source of data on injured patients was the trauma registry of England and Wales, TARN based at the University of Manchester. Data covering the 10 year period January 1, 1996 to December 31, 2005 were included.

Since its establishment in August 1989 175 Accident and Emergency units in England, Wales and Northern Ireland (62% of the total of 281 A&E units in 2005) have voluntarily reported to TARN [12]. At the time of analysis TARN database contained over 215 000 cases. Eligible cases are:

• trauma admissions to participating hospitals with length of stay of 72 hours or more,

• trauma admissions to an intensive care or high dependency area,

• deaths from injury after arriving alive at hospital,

• transfers to another hospital for specialist care.

Based on international trauma registry criteria, the exclusion criteria include:

• isolated fractures of the femoral neck or single pubic rami, in patients 65 years or more,

• uncomplicated spinal sprains,

• closed facial injuries,

• simple skin injuries [13].

The TARN dataset comprises patient age, sex and postcode of residence – socio-demographic characteristics, circumstances of the incident, description of the injury(ies) based on the Abbreviated Injury Scale, healthcare details and vital signs as well as clinical outcome (alive/dead at hospital discharge or 30 days whichever is sooner). Injury Severity Scores are calculated by TARN staff. TARN variables used in this study were age, year of admission, patient and incident postcode (place of residence and injury occurrence respectively), transfer status, injury code (based on Abbreviated Injury Scale (AIS)-98), hospital network code and postcode [14,15].

TARN applies a quality assurance programme that includes:

-validation during all steps of data input (e.g. returning/resubmission of data collection forms for reason related to eligible criteria or missing obligatory data – i.e. hospital identification number, trauma network number, date of birth, sex, type and cause of injury, date of arrival at hospital, outcome, date of death or discharged, and at least one injury description),

-injury coding verification through reliability studies (reanalysis of patient records),

-parallel data processing – new database programs under development.

Moreover, hospitals are asked to check the accuracy and completeness of the data submitted (e.g. special attention given to completeness of the fields, recording of dates or digit within codes), to find out trauma patients meeting the TARN eligible criteria within the other wards from the hospital (if applicable) and to submit the data on the dedicated forms [14].

#### (2) Postcode data

In UK there are 124 postcode areas and each area is split up into districts, sectors, sub-sectors and units. The postal code (postcode) is alphanumeric, up to 7 characters long; the first two (or four) characters identify the major area and the corresponding district, and the last three characters representing the address, usually within 80 properties. Although the TARN database contains full postcodes this is not a mandatory variable so the completeness of this item varies. Therefore we used district postcode (i.e. first part of postcode) since completeness was generally good.

#### (3) HES data

Additionally, to validate rates computed using TARN data we used Hospital Episode Statistics over eight financial years, i.e. 1996/1997 to 2003/2004. HES is the national statistics data source for England that relies on hospital admissions data submitted by NHS Trusts to the NHS-Wide Clearing Service. It includes a large number of fields such as patient age, sex and district postcode, admissions and discharges, episodes and spells, diagnoses, organizations [16]. HES variables used in this study were year of admission, duration of spell, patient district postcode, the code of injury – primary diagnosis 3 character (based on ICD-10^th ^revision), and the hospital code.

It is noteworthy HES categorizes injuries using around 800 definitions (ICD-10^th ^revision, S00-T32); data do not differentiate injuries in terms of severity using ISS or any other specific measure of severity whereas TARN database lists over 1200 serious injuries (AIS) and differentiates overall anatomical injury insult by severity (ISS).

#### I. Identifying the injured patient numerator – selection of hospitals from the TARN database

Our aim was to develop a method for constructing catchment areas so that we can calculate population-based injury occurrence rates. The TARN database contains postcodes since 1996 (when the data collection forms were improved to include more variables). We therefore selected hospitals that have reported continuously from 1996–2005, each with overall >70.0% completeness of postcodes. We then selected those hospitals with *local market areas*, defined as drawing >70% of their patients from the same *postcode area *in which the hospital was located.

Database quality was examined using yearly admissions reported to TARN by each selected hospital, the completeness rates for the variables of interest. For one selected hospital, i.e. North Staffordshire Royal Infirmary (NSRI), we performed a cross-check between TARN and a secondary source based on mandatory reporting, i.e. Hospital Episode Statistics. An exhaustive option to determine completeness – i.e. medical record review – was not viable for logistical reasons. NSRI is a tertiary care centre with the highest yearly attendance rate and, consequently, the highest number of cases reported per year, by comparison with other TARN hospitals.

Data from HES and TARN were extracted so that they met common admission criteria. Cases with hospital stays of three days or under and ICD codes representing minor injuries (sprains) were removed from the HES database. TARN data were re-categorized by financial year rather than calendar year. Since HES – based on the 3 character ICD – does not distinguish fracture of the neck of femur from all other femoral fractures and TARN excludes the former in people 65 years and older, all fractures of the femur were removed from both databases to allow better case comparison. The capture rate of the TARN database was defined as the yearly proportion of the HES cases that were detected by TARN, expressed as a percentage at NSRI.

#### II. Identifying the denominator population – delineation of hospital catchment areas

We selected the hospitals with >70% completeness of patient postcodes and a defined local market area. We employed a 'variable market approach', similar to that used in the United States to assess competition between hospitals, to allow the geographical area assigned to each hospital to vary according to unit characteristics [17]. One feature of this method is based on *patient flow*, defining and including in further analysis the standard geographical areas (in our study the postcode districts) that send a significant number of patients, collectively accounting for 40–95% of hospital discharges [18,19].

#### (a) Describing the exact catchment area

Firstly, after excluding hospitalisation of out-of-area residents, all the trauma patient district postcodes within the postcode area of the given hospital were arranged in decreasing order of frequency. Secondly, we determined the district postcodes that cumulatively accounted for a threshold of significance, defined in our study as a minimum of 80% of the hospital's local patients. The selected postcodes were mapped to demarcate the market area and to assure contiguity, i.e. contiguous postcode districts groups with no gaps.

#### (b) The impact of competitor hospitals

An important issue in defining hospital catchment areas was the identification of competitor hospitals for the same pool of patients since this may influence the delineation of catchment areas. All hospitals located within a 15 mile radius of the hospital under study in urban areas (30 miles in rural areas) might be potential competitors [20-22]. In our study we used a 20 miles radius to identify competitors – a compromise between these two figures for urban/suburban populations. If no information was available from TARN database concerning trauma admissions to competitor hospitals, asymmetry of competition and the road travel map were examined with the purpose of avoiding overlap between market areas. Asymmetry of competition refers to larger hospitals that may be competitors for smaller district hospitals, without the reverse being true. In the final analysis we selected only the hospital catchment areas that had no major competitors, either from the TARN database or using additional information (maps/hospital size).

Hospital catchment areas have been characterised by the number of hospitals per area; the average number of potential competitors per area; average number of districts per area; area size; and by the admissions of the catchment area residents to the hospital(s) located inside/outside hospital catchment area.

To check the robustness of this patient flow approach we employed a 'patient origin matrix', as an alternative option to construct the market area for one selected hospital – NSRI, Stoke on Trent [23,24]. We used the HES database as an additional data source on hospitals in the region that are not part of TARN network. Each cell gives discharges of patients from a particular hospital that are resident within a particular district. Consequently, we established the hospital with the highest number of discharges per district – the main hospital provider for each district and consequently the market area for NSRI. We compared this with the market area derived from TARN data.

#### Ethical approval

The TARN database stores no patient identifiers. Approval for research using TARN anonymized data is granted by the Patient Information Advisory Group (PIAG Section 60) and supported by the Healthcare Commission.

## Results

### The injured patient numerator

Sixteen TARN hospitals out of a total of 41 hospitals meeting the initial selection criteria (continuous membership over 1996–2005 and over 70% completeness of postcodes) were found to have appropriate numerator data for trauma burden. The characteristics of these hospitals, hospitals not part of TARN and hospitals with A&E units in England, Wales and Northern Ireland by size of unit and location are shown in Table 1. Although the 16 hospitals included in this study cover the range of yearly attendances, there are no hospitals in London, Wales or Northern Ireland.

**Table 1:** Characteristics of the A&E units in England, Wales & Northern Ireland, and of TARN hospitals

	**All hospitals with A&E units**	**All hospitals TARN members over time**	**Hospitals under study**
**Yearly attendances (emergency room)**			
<30000	56	13	1
30000–59999	125	87	7
60000–89999	81	62	4
= 90000	19	13	4
**Location**			
London	40	15	
Midlands & Eastern	61	38	8
North	83	65	7
South	61	42	1
Wales	19	12	
Northern Ireland	17	3	

**Total**	**281**	**175**	**16**

Source: Data Directory of Critical Care 2005 Cambridge, CMA Medical Data 2005

An overview of the number of cases reported annually by the hospitals in this study and the completeness of patients' postcodes of residence is given in Table 2. To avoid bias resulting from analysing data from years with large amounts of missing data we excluded those years with large amounts of missing postcodes values (under 70% completeness rate). Also, since there is up to one years delay in reporting cases for the previous calendar year admissions during the year 2005 were excluded. Some years appear low in particular hospitals; these years do not contribute to calculation of annual incidence rates.

**Table 2:** Number of reported cases per year by selected TARN hospitals, 1996–2005

**Number of cases (% completeness rate of patient's residency postcodes)**										
**TARN hospital**	**1996**	**1997**	**1998**	**1999**	**2000**	**2001**	**2002**	**2003**	**2004**	**2005**

**North Staffordshire Royal Infirmary**	**980 (56.2)**	867 (94.8)	841 (98.5)	869 (96.4)	*488 *(94.2)	754 (93.3)	674 (95.1)	*441 *(95.4)	*191 *(95.8)	**76 (94.7)**
**Nottingham University H**	**931 (69.7)**	857 (89.1)	781 (92.8)	890 (97.4)	736 (97.0)	673 (93.0)	883 (97.3)	726 (98.2)	834 (98.1)	**34 (100.0)**
**The Ipswich H**	264 (87.9)	279 (93.2)	267 (95.5)	301 (98.7)	292 (98.9)	295 (98.3)	317 (99.7)	371 (99.2)	327 (99.1)	**172 (99.4)**
**Leicester Royal Infirmary**	501 (96.8)	501 (99.2)	449 (99.8)	560 (98.4)	723 (99.2)	528 (99.2)	370 (100.0)	*246 (*100.0)	*249 (*100.0)	**77 (100.0)**
**Colchester General H**	206 (85.9)	237 (97.5)	230 (98.7)	260 (98.1)	269 (95.9)	139 (98.6)	149 (90.1)	*121 *(84.3)	*35 *(85.7)	**16 (81.2)**
**Northampton General H**	*46 *(95.6)	260 (96.9)	266 (98.5)	*191 *(97.9)	*17 *(100.0)	*5 *(100.0)	*55 *(94.5)	367 (100.0)	430 (100.0)	**222 (99.5)**
**West Cumberland H**	86 (73.3)	62 (100.0)	67 (94.0)	65 (96.9)	101 (97.0)	83 (98.8)	68 (100.0)	73 (100.0)	65 (98.5)	**12 (100.0)**
**Scunthorpe General H**	**208 (1.4)**	**175 (4.6)**	175 (78.8)	156 (98.1)	146 (99.3)	159 (98.7)	169 (98.8)	210 (99.5)	141 (100.0)	**6 (100.0)**
**Huddersfield Royal Infirmary**	**85 (5.8)**	**95 (12.6)**	**81 (48.1)**	100 (91.0)	91 (96.7)	*68 *(94.1)	128 (92.2)	148 (89.2)	201 (96.5)	**78 (88.5)**
**Peterborough District H**	352 (87.2)	283 (96.8)	263 (97.7)	278 (98.6)	312 (98.4)	*201 *(96.5)	*144 *(92.4)	*179 *(96.6)	*154 *(98.7)	**0**
**Pilgrim H**	145 (71.0)	130 (89.2)	179 (80.4)	187 (95.7)	176 (97.2)	191 (98.4)	273 (94.5)	237 (96.2)	219 (98.6)	**21 (95.2)**
**Royal Liverpool University H**	*122 *(74.6)	*138 *(99.3)	*116 *(94.8)	*93 *(98.9)	272 (98.9)	284 (98.6)	309 (99.0)	359 (98.6)	353 (98.3)	**32 (100.0)**
**University H Aintree**	*182 *(87.4)	*181 *(97.2)	*167 *(97.6)	*218 *(94.0)	288 (96.5)	280 (97.9)	308 (99.4)	342 (99.7)	*106 *(100.0)	**0**
**Countess of Chester H**	350 (79.4)	297 (96.6)	283 (94.7)	308 (97.1)	320 (98.8)	376 (99.2)	327 (98.5)	351 (99.4)	302 (98.7)	**4 (100.0)**
**Arrowe Park H**	413 (84.3)	394 (97.0)	372 (94.1)	330 (94.5)	304 (97.7)	415 (94.9)	396 (95.7)	300 (98.0)	*287 *(99.3)	**5 (100.0)**
**Poole H**	478 (80.5)	521 (93.7)	345 (93.6)	*53 *(100.0)	332 (97.3)	*235 *(99.1)	**-**	*130 *(95.4)	*113 *(97.4)	**0**

- data not available; data in bold represent years excluded from defining hospital catchment areas; italics indicate low data returns which will not contribute as numerator for the corresponding year in the future computation of injury rates.

### Numerator capture rate analysis – data quality

The results of cross-checking TARN data and HES returns for NSRI, shows a numerator capture rate of 73.4% (5058 TARN cases vs. 6895 HES cases) (Table 3). Capture rates in 2003/4 and 2000/01 were low and were therefore excluded from the calculation of trauma incidence rates. Removing these years from the analysis gives an adjusted capture rate of 83.5% (4342 TARN cases vs. 5194 HES cases).

**Table 3:** Capture rate for all major trauma injuries (excluding fracture of femur) by year, NSRI Stoke on Trent, 1996–2004

**Year**	**HES cases**	**TARN cases**	Capture rate (%) *
1996/1997	714	835 (714**)	100.0**
1997/1998	987	789	80.0
1998/1999	939	768	81.8
1999/2000	971	772	79.5
2000/2001	943	421	44.6
2001/2002	891	667	74.9
2002/2003	692	632	91.3
2003/2004	758	295	38.9

**Total**	**6895**	5058**	**73.4**

Source: Trauma Audit and Research Network & Hospital Episode Statistics (Health and Social Care Information Centre)

*expressed as percentage of yearly number of HES cases that were detected in TARN database

** after correction for overreporting made by excluding 121 cases from 835 TARN cases in 1996/1997 apparently not admitted to NSRI according to HES data but which appeared in the TARN database

### The denominator population

#### 1. Determining the NSRI catchment area

**Figure 1:**
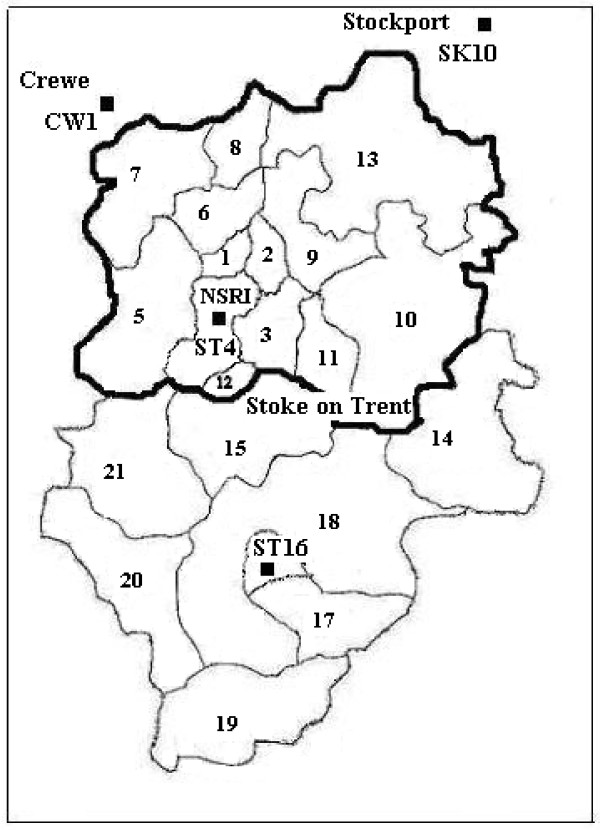
Map of Stoke on Trent area with numbers indicating the districts within Stoke on Trent area, dots representing NSRI and the hospitals within a 20 miles radius (labelled according to the postcode district location), and the hospital catchment area for NSRI delineated in black.

We present a detailed example to show how we applied the market area technique to determine the catchment area for NSRI, in Stoke on Trent (Table 4). In line with our a priori criteria a high percentage of postcodes were available (i.e. 95.6%, 4898/5125) and over 70% of patients discharged from the hospital NSRI originate from the same area where the hospital is located – Stoke on Trent (i.e. 75.8%, 3886/5125). Table 4 also shows patient's postcodes of residence by postcode districts in descending order of frequency, after removing from the database the patients who lived out of the area as well as all admissions during 1996 (56.2% completeness rate for postcodes) and 2005 (delay in data reporting). In bold are the postcode districts that account for the threshold of 80% of hospital's admissions: ST1 to ST7, ST10 and ST13. We then visualised these districts on map and delineated a contiguous catchment area (Figure 1).

**Table 4:** Patient's residence – area postcodes (all selected hospitals) and – district postcodes after removing out-of-area cases (NSRI, Stoke on Trent 1997–2004)

	**Patient postcode-all hospitals**					Patient postcode – NSRI***		
	
**Hospital (s)/area postcode, time period**	**Hospital area No (%)**	**Out-of-hospital area No (%)**	**Not known No (%)**	**Total No (100.0%)**		**ST district postcode**	**Total **(area + not known)	
							
							**No**	**(%)**
						
				
**North Staffordshire Royal Infirmary/ST, 1997–2004**	3886 **(75.8)**	1012 (19.8)	227 (4.4)	5125	→	**ST5**	613	**14.9**
					**→**	**ST6**	533	**13.0**
						**ST3**	533	**13.0**
**Nottingham Univ. H/NG, 1997–2004**	4661 **(73.1)**	1425 (22.3)	294 (4.6)	6380		**ST4**	456	**11.1**
						**ST2**	312	**7.6**
**The Ipswich H/IP, 1996–2004**	2344 **(86.4)**	287 (10.6)	82 (3.0)	2713		**ST7**	288	**7.0**
						**ST1**	289	**7.0**
**Leicester Royal Infirmary/LE, 1996–2004**	3528 **(85.5)**	559 (13.5)	40 (1.0)	4127		**ST10**	211	**5.1**
						**ST13**	182	**4.4**
						ST8	125	3.0
**Colchester General H/CO, 1996–2004**	1296 **(78.7)**	256 (15.6)	94 (5.7)	1646		ST11	72	1.8
						ST9	66	1.6
**Northampton General H/NN, 1996–2004**	1395 **(85.2)**	221 (13.5)	21 (1.3)	1637		ST15	42	1.0
						ST14	33	0.8
						ST16	28	0.7
**West Cumberland H/CA, 1996–2004**	550 **(82.1)**	86 (12.8)	34 (5.1)	670		ST12	24	0.6
						ST17	21	0.5
**Scunthorpe General H/DN, 1998–2004**	1031 **(89.2)**	79 (6.8)	46 (4.0)	1156		ST18	17	0.4
						ST21	13	0.3
**Huddersfield Royal Infirmary/HD, 1999–2004**	592 **(80.4)**	95 (12.9)	49 (6.7)	736		ST19	8	0.2
						ST20	5	0.1
						ST	15	0.3
**Peterborough District H & Pilgrim H/PE, 1996–2004**	2972 **(76.1)**	702 (18.0)	229 (5.9)	3903		Not known	227	5.5
						
						**Total**	**4113**	**100.0**
						
Royal Liverpool H, Univ. H Aintree, Countess of Chester, Arrowe Park H*/L+CH, 1996–2004	8772 **(85.6)**	1053 (10.3)	418 (4.1)	10243				
Pool H/BH, 1996–2004**	1705 **(77.3)**	334 (15.1)	168 (7.6)	2207				

*all cases age ≥ 16 years; ** excludes year 2002; *** in bold the first districts that account for **83.1**% of hospital admissions

The potential competitors for NSRI inside a 20 miles radius are Leighton Hospital – CW1 (13.8 miles), Staffordshire General Hospital – ST16 (14.2 miles) and Macclesfield District General Hospital – SK10 (17.8 miles) (Figure 1). Although Leighton Hospital was a member of TARN for five years, and the registry shows that it draws significant numbers of patients from ST7, it was not the 'main provider' for this shared district (44 cases Leighton Hospital vs. 216 cases NSRI, 1996–2000). Staffordshire General Hospital and Macclesfield District General Hospital have never submitted TARN data. However, yearly attendances of NSRI vs. these hospitals show an asymmetry of competition (96 000 vs. 56 016, respective 96 000 vs. 36 000). Additionally we analysed the map for major roads that traverse the region – they did not separate our proposed catchment area for NSRI. Therefore, we have designated a catchment area for NSRI as outlined in the bold black line on Figure 1, comprising post code districts ST1–13.

### Sensitivity analysis – patient origin matrix

Table 5 shows the main provider of trauma care services for residents of each of the ST districts. It suggests that NSRI is the main provider of trauma care for the residents ST1-ST13. This equates with the areas defined by the 80% cut-off from TARN data; ST8, ST9, ST11 are the next districts in descending order of frequency, up to 89.5% whereas ST12 has been added for geographic contiguity (Figure 1). It is noteworthy that whereas patient flow concerns a proportion of each hospital's local patients (in this study defined by a minimum of 80%), the patient origin matrix allows delineation of the entire hospital market area. Apart from NSRI area the patient origin matrix shows the catchment area for Staffordshire General Hospital (i.e. ST15 to ST21) with one competitor and main provider for the district ST14, which is Queen's Hospital.

**Table 5:** Patient origin matrix for NSRI and the hospitals in the region understudy labelled according to the postcode district location (data in bold represent cases per district corresponding to the hospital main provider)

	**All the postcode districts within ST postcode area**																				
**Hospitals in the region**	**ST1**	**ST2**	**ST3**	**ST4**	**ST5**	**ST6**	**ST7**	**ST8**	**ST9**	**ST10**	**ST11**	**ST12**	**ST13**	**ST14**	**ST15**	**ST16**	**ST17**	**ST18**	**ST19**	**ST20**	**ST21**

**ST4**	**379**	**459**	**737**	**645**	**968**	**822**	**453**	**203**	**122**	**291**	**107**	**37**	**290**	22	61	40	28	15	10	9	19
**ST16**	-		9	9	-	-	-			10	5		-	43	**153**	**231**	**344**	**140**	**113**	**37**	**56**
**SK10**	-	-	-	-	-	-	14	12	-	6		-	21	-							
SK2*					-	-							-			-		-			
CW1*	-	-	-	-	5	-	44	-						-							-
TF1*							-											-		-	
**WV10**	-															-	-		15		
**WS2**			-		-	-												-	-		
DE13*		-	-			-			-	-	-	-		**142**	-	-					-
**DE1**														-	-	-		-			

Source: Trauma Audit and Research Network & Hospital Episode Statistics (Health and Social Care Information Centre)

e.g. 737 cases with residence ST3 were admitted to NSRI – the highest number in the column, therefore NSRI is main ST3 provider – cells with under 5 cases suppress (data protection requirements)

*data over shorter time periods then 1997–2004, depending on the number of years TARN membership per hospital NSRI, Stoke on Trent (**ST4**), Staffordshire General Hospital, Stoke on Trent (**ST16**), Macclesfield District General Hospital, Stockport (**SK10**), Stepping Hill Hospital, Stockport (**SK2**), Leighton Hospital, Crewe (**CW1**), Princess Royal Hospital, Telford (**TF1**), New Cross Hospital, Wolverhampton (**WV10**), Manor Hospital, Walsall (**WS2**), Queen's Hospital, Derbyshire (**DE13**) and Derbyshire Royal Infirmary (**DE1**)

If we define the competitors as hospitals that compete notably for the patients from at least one of the districts that are part of the market area, from the cross tabulation it can be observed hospitals that compete for NSRI catchment area patients are Staffordshire General Hospital (10 cases ST10, 5%), Leighton Hospital (44 cases ST7, 13.2%) and Macclesfield District General Hospital (14 cases ST7 – 4.2%, 12 cases ST8 – 9.2% and 21 cases ST13 – 11.1%). The percentages in the brackets represent market share – admissions from the corresponding district, and are approximate values since the database includes hospitals that report cases over different time periods from 1996 to 2004. The hospitals mentioned above are also those that fall within a 20 mile radius of NSRI, the other ones being located up to 31.2 miles radius (i.e. Derbyshire Royal Infirmary).

#### 2. Delineation of hospital catchment areas for the selected TARN hospitals

Using the variable market area approach described above the hospitals selected represent 12 denominator catchment areas: ten one-hospital areas, one two-hospital area and one four-hospital area. The catchment areas of two hospitals from the Peterborough area were merged into one larger area; hospital catchment areas of four selected hospitals located in Liverpool and Chester were merged into one area since residents of the Wirral Peninsula (i.e. 15 districts) had their postcodes changed at the middle of the study period from L (Liverpool) to CH (Chester). This last area excludes patients under 16 years of age to eliminate one potential competitor dealing with paediatric trauma, for which no TARN information was available.

A total of 40543 admissions were used to delineate hospital catchment areas; local market areas were expressed by a percentage that ranged from 73.1 to 89.2%, i.e. proportion of patients resident within postcode area of the hospital location (Table 4). The number of each hospital potential competitors ranged from zero to 13.0. On average there were 3.5 potential competitors per defined catchment area. From a total of 41 potential competitors, 23 fall within a 15 miles radius. Information was available from the TARN database for 29 (out of the 41) competitor hospitals. Two hospitals were minor injury units, one hospital does not receive trauma patients, nine hospitals were assessed as irrelevant by asymmetry of competition and visualisation of road travel maps.

From the patient flow process to identify catchment area ten out of 12 catchment areas have been reassigned for contiguity, two areas after map visualization. No catchment area has been reassigned based on competition for the same pool of patients. The number of districts per catchment area ranged from 7 to 40 (average 15.1). Area size was expressed by a minimum/maximum linear distance from the hospital to the area borders bearing in mind that there is no assumption of a circular shape. The averages were 4.3(2.8–5.8)/18.1(15.8–20.4) miles. Larger areas were seen in regions surrounded partly by water. Table 6 displays total admissions of the catchment area residents, i.e. those to the hospital(s) located inside as well as outside delineated hospital catchment area.

**Table 6:** Major trauma admissions of catchment area residents to the hospitals located inside (admissions internal) and outside (admissions external) catchment area

Hospital (s), time period (hospital catchment area*)	**Total admissions**	**Admissions internal %**	**Admissions external%**
**Royal Infirmary Stoke on Trent, 1997–2004 (ST1–13)**	3812	97.2	2.8
**Nottingham Univ. Hospital, 1997–2004 (NG1–12, NG16)**	4274	94.5	5.5
**The Ipswich Hospital, 1996–2004 (IP1–17)**	2280	97.3	2.7
**Leicester Royal Infirmary, 1996–2004 (LE1–9, LE11, LE12, LE18, LE67)**	3470	91.0	9.0
**Colchester General Hospital, 1996–2004 (CO1–16)**	1508	85.9	14.1
**Northampton General Hospital, 1996–2004 (NN1–7, NN11)**	1281	93.3	6.7
**West Cumberland Hospital, 1996–2004 (CA13–15, CA22–28)**	528	95.3	4.7
**Scunthorpe General Hospital, 1998–2004 (DN14–18, DN20, DN21)**	1259	71.5	28.5
**Huddersfield Royal Infirmary, 1999–2004 (HD1–5, HD7, HD8)**	633	86.9	13.1
**Peterborough District Hospital & Pilgrim Hospital, 1996–2004 (PE1–12, PE15, PE20–22)**	2841	91.1	8.9
**Royal Liverpool Univ. Hospital, Univ. Hospital Aintree, Countess of Chester, Arrowe Park Hospital^1^, 1996–2004 (L1–13, L15, L17, L19–21, L30, CH1–5, CH41–49, CH60–66)**	7790	96.8	3.2
**Pool Hospital, 1996–2004^2 ^(BH1–25, BH31)**	1791	94.5	5.5

*postcode districts

^**1**^all cases age ≥ 16; ^**2 **^excludes year 2002

## Discussion

In an ideal world, for calculating population-based rates, full patient postcodes would be available from all hospitals within larger areas to give an accurate description of the numerator and hospital catchments would be well defined and understood so that denominators were similarly well-defined. Unfortunately this is not the case. This paper offers an approach to defining hospital catchment areas so that population-based rates can be calculated, using information from a trauma registry, TARN database. Its value is that, potentially, any large high quality national trauma database could provide incidence rates upon which injury control programmes can be based.

From the point of view of an 'injury pyramid', we excluded minor injuries, i.e. injuries other than those resulting in more severe or fatal outcomes, as these are not generally included in trauma registries. A high proportion of minor injuries result in self-care and/or no medical treatment and any incidence estimates are therefore difficult to obtain, except through special surveys. Although we recognise that minor injuries have a substantial impact on morbidity or health care utilization of services [25], major injuries are those generally requiring much attention in terms of control programmes (either prevention, acute care or rehabilitation) if 'savings lives' or preventing long term disability is the final goal.

### Hospital market areas

In economics a market is an area where sellers and buyers interact to establish prices. In the health care field the 'market area' concept has been extensively used in north America, by researchers interested in measuring the intensity of competition between hospitals (i.e. Hirschman – Herfindahl index) and utilization of health services. Hospital administrators also use this tool to define the competitors in the region, as do policy makers willing to control hospital prices. More recently Primary Care Service Areas have been established for assessing primary care services [17,20,26]. The notion of market area (Hospital Service Area – defined by means of patient origin matrix) has been recently employed by researchers in Switzerland to describe utilization of health care services [24] whereas in the UK, Propper et al. have used market areas (defined by patient travel time) to look at the relationship between quality and competition in the UK health care system [27].

Although never used before in an epidemiological analysis we believe that market area- as defined by our methodology – can provide a means of determining denominators for calculating major injury rates based on datasets from any large national trauma registry. In this context it is noteworthy that ISS was also initially developed within the framework of medical audit and afterwards used in epidemiology for its ability to identify patients with severe injury, overcoming selection bias [28] and reducing the potential for epidemiological analyses confounded by injury severity. (Severity of injury would always increase probability of admission irrespective of other factors such are bed supply, hospital admission policies, socio-economic status of the patient.)

In this study the estimates of hospital catchment areas – subject to data quality – cover time intervals that vary from 6-year (1999–2004) for Huddersfield Royal Infirmary up to 9-year (1996–2004) for most of the hospitals. Although these areas depend on population usage of services that might vary from one year to another, we believe that the delineation of areas would not be significantly changed if constructed over longer time periods. To test this assumption we reassessed the hospital catchment area for NSRI in two 4-year windows (i.e. 1997–2000 and 2001–2004). This showed a similar rank order for the first districts that account for up to 80% of the hospital local admissions and consequently no differences in the area delineation. Moreover, to find out the extent to which the 20 mile radius was sufficient to identify potential competitors, we examined distances travelled to TARN hospitals by all modes of arrival, based on a randomly selected sample of 100 patients admitted to all TARN hospitals over 1996–2005, with the exception of the units located in London, Northern Ireland and Wales for which there is lack of representativeness. Patients travelled an average distance of 4.7 (3.7–5.7, 95%CI) miles (range 0 to 31.0; median 2.9 miles). These results as well as the NSRI market area constructed from the HES/TARN patient origin matrix supports our approach as relatively robust.

### Limitations

A notable limitation is that not all A&E Department in the UK report to TARN. Furthermore, those that do report voluntarily submit data of varying quality. Therefore our sample of hospitals is a convenience sample rather than a representative one. There are no units included from London, North Ireland and Wales which limits the generalizability of population-based rates to England. In relation to the size of sample, the analysis uses 16 hospitals out 245 receiving trauma in England, i.e. a reasonable 7% sample. A mix of urban (4/16) and rural, teaching (5/16) and general hospitals submit to TARN; this reflects the mix of types of hospitals receiving trauma. In addition our further work with census data show our population resident within delineated areas is representative of that of England outside London (data not shown).

The majority of the mandatory variables (such as age, sex, cause of injury or transfer status) in the TARN database are 100% complete (data not presented in the paper). A notable exception is patients' postcodes of residence, which is not compulsory for registration, and reporting is incomplete. To overcome this we decided to create market areas (and consequently to report rates) using hospital discharges from years with reasonable data quality. An additional issue related to this item was the format of the postcode. Using district level postcodes as we have done used limits pinpoint accuracy in determining market areas. However, we believe that district postcodes geographically aggregated are a practical alternative, and other researchers have used census districts, which comprise aggregated zip codes [24].

In addition, since we were working with aggregate postcodes instead of zip codes, in the patient flow analysis we decided not to set out a marginal value to define patients that individually contribute at least 1% – 3% of hospital discharges [18,19]. However, a retrospective analysis of the delineated catchment areas in this study gave a marginal value of 0.6%.

The number of trauma cases reported per year for each selected hospital shows large year on year fluctuations (Table 2, figures in italics). Although this might reflect true changes in major trauma occurrence, it is likely to be a surveillance artefact. TARN is a voluntary reporting scheme and hospitals can opt in or out of the scheme at any time. Personnel changes can affect the timeliness with which data are submitted and data quality. This is why we decided to exclude years of apparently low reporting from further analysis of estimating population-based rates. Of note data quality does not appear to vary by hospital type. Supporting this decision is the outcome of the capture rate analysis for NSRI against a mandatory reporting database (i.e. HES) that shows an overall rate of 73.4% with as little as 38.9% or 44.6% coverage for years that demonstrate significant decreases in the number of reported cases. In this context it should be noted the results of a sensitivity analysis (based on medical records review) for the United States National Electronic Injury Surveillance System over three months in 1990 showed an overall sensitivity similar to that in out study [29]. However, in reporting rates we will just be using years of good data reporting where the maximum underreporting can be estimated to be under 16.5% from our comparison with HES. The data quality for several of our study hospitals is consistently high (Table 2 and through TARN quality assurance). We therefore do not anticipate our underreporting rate will exceed 10% which will allow useful injury control initiatives and international comparisons.

### Population-based rates (taking account of out-of-area hospitalisations)

In this study we defined hospitals catchment areas so that we can go on to construct population-based rates of injury, the numerator being total admissions of residents within a particular catchment area and the denominator being the population resident within that catchment area. Since some patients that sustain injury might be admitted to a hospital outside their residential area this cross border flow might cause problems when computing rates. Therefore we chose an approach designed to minimise patient outflow (i.e. over 70% of the patients originated from the same postcode area where the study hospital was located) in order to minimize numerator-denominator mismatch [30]. However, to account for the inpatients that live in one of the defined areas but receive medical care in another, when computing incidence rates the numerator will include all these cases based on a reassignment procedure (Table 6). It is possible that – without data input – our numerator might underestimate the true occurrence of injury events in selected areas because some people resident in one hospital catchment area may be treated in another hospital that is not part of TARN database. Since hospitalisations of area residents have been counted without regard to transfer status, in order to avoid duplicates, inter-hospitals transfers of these reassigned cases were discounted if the transfer was from/to the hospital main provider for the corresponding hospital catchment area. The TARN database excludes repeat admissions for the same injury, therefore allowing estimation of major injury rates based on hospital admissions.

## Conclusion

Large national trauma registries, and in particular TARN, hold data that have the potential for calculating population-based injury rates. Although similar populations with regard to place of residency and hospital admission are highly desirable in estimation of rates, we believe that our approach may be used to assess injury occurrence within regions characterized by low patient movement.

## Abbreviations

A&E (Accident & Emergency); AIS (Abbreviated Injury Scale); International Classification of Diseases (ICD); ISS (Injury Severity Score); HES (Hospital Episodes Statistics); NSRI (North Staffordshire Royal Infirmary); SES (socioeconomic status); TARN (Trauma Audit and Research Network)

## Competing interests

The author(s) declare that they have no competing interests.

## Authors' contributions

RA and FL designed the study; RA performed the analyses and drafted the manuscript; FL contributed to the written manuscript and supervised the study; SO and RL provided feedback on the manuscript and advised on the analyses. All authors read and approved the final manuscript.

## Pre-publication history

The pre-publication history for this paper can be accessed here:


